# Ethnobotanical study on plants used to dye traditional costumes by the Baiku Yao nationality of China

**DOI:** 10.1186/s13002-021-00497-2

**Published:** 2022-01-04

**Authors:** Renchuan Hu, Tingting Li, Yunrui Qin, Yujing Liu, Yunfeng Huang

**Affiliations:** 1 Guangxi Key Laboratory of Traditional Chinese Medicine Quality Standards, Guangxi Institute of Traditional Medical and Pharmaceutical Sciences, Nanning, 530022 China; 2 Jiangbin Hospital of Guangxi Zhuang Autonomous Region, Nanning, China; 3 College of Life Sciences, Nanjing Agriculture University, Nanjing, 210000 China

**Keywords:** Ethnobotany, Clothing dyeing plant, Guangxi and Guizhou province, Ethnic culture, Traditional knowledge

## Abstract

**Background:**

The Baiku Yao is a branch of the Yao nationality in China. The unique dying process of traditional clothing employed by these people has distinct national characteristics, a profound impact on the production and life of local people, and important research value. For this reason, it is important to investigate and document the dyeing plants and the traditional knowledge involved in the dyeing of Baku Yao traditional clothing.

**Methodology:**

Information on dyeing plants was obtained using the free-list method and interviews with 61 Baiku Yao informants in Guangxi and Guizhou from January 2020 to August 2021. Based on the free-list method, we evaluated and screened out important plants by calculating the cognitive salience value of each plant.

**Results:**

The results showed that the people of Baiku Yao have accumulated rich traditional knowledge of dyeing plants and long-term dyeing and other processes. We collected 23 species and recorded the related traditional knowledge, such as the Baiku Yao name, life form, habitat, part(s) used, application type, usage, and area used. The utilization of dyeing plants has a strong relationship with ethnic culture. The Baiku Yao uses unique anti-dyeing material (*Ailanthus vilmoriniana*) in the cotton dyeing process, they call it “the mother tree”. As well as, the results also showed that 15 plants (65.2%) have medicinal value and can be used to treat 18 aliments, and wild plants play a very important role in the life of the Baiku Yao.

**Conclusion:**

These plants not only meet the color needs of the Baiku Yao people but also have health care effects, aligning with the trends toward green dyeing and the health contentiousness of modern people. This study provides a reference for the inheritance and development of Baiku Yao traditional clothing dyeing culture, will aid the identification of new natural, safe and healthy textile dyes, and is of positive significance in promoting regional economic development, national cultural inheritance, and biodiversity protection.

## Introduction

Plant dyeing refers to the process of extracting pigments from plants and combining pigments with fabric through direct or mordant dyeing [1]. As a natural dyeing technology, plant dying echoes the contemporary demands for green and healthy products. Natural materials can not only meet the needs of providing rich color but are also easy to obtain, biodegradable and cause little environmental pollution. Many dyeing plants also have certain medicinal and health care functions, which are of great research, and promotion value [2].

Chinese people have a long history of using plant-originated dyestuffs, and traditional dyeing plant resources are rich, especially in many remote mountain villages [3]. As early as 4500 years ago in the period of the Yellow Emperor, people began to use plants for dyeing [4]. However, the few color types, easy fading, and long process of extracting dyes from plants limit their popularization and application [5]. With the development of modern science, chemical synthetic dyes have gradually replaced traditional plant dyes and become the main coloring material of textile dyeing because of their advantages, such as complete chromatography, bright color, washing and sun resistance, and low price. With the enhancement of people's awareness of environmental protection, the irritation of chemical dyes to the skin and their contribution toward pollution is gradually being recognized. Traditional plant dyes not only come from convenient materials and provide unique colors but can also satisfy people's pursuit of personalization and diversification while being biodegradable and not polluting the environment. In addition, many of these plants have medicinal and food value, with healthy effects on the body, which has increased awareness of the unique advantages of these dyeing plants [6–8].

Some ethnobotanical research on dye plant has been carried out, such as Bai, Dai, Buyi, Zhuang [3, 9–12]. The Baiku Yao is one of many branches of the Yao nationality. The Baiku Yao is an ancient nation with unique clothing culture. In long-term agricultural production and labor, the Baiku Yao people have created unique weaving skills, and traditional plant dyeing techniques have been retained. This group is a very little-known minority, and its members call themselves "Duo nu". In China, clothing is the best way to identify an ethnic minority. This nationality is named Baiku Yao because the men usually wear white pants. White pant culture not only refers to the white pants worn by men but also includes the pleated skirts worn by women. There are both festival costumes and ordinary clothes. Women's clothes are also divided into winter clothes and summer clothes. Baiku culture plays a leading role in the development of the Baiku Yao and determines various differences in religious beliefs, production conditions, personality, and psychology, which constitute the characteristics of Baiku Yao culture and differentiate between Baiku Yao and other Yao branches [13]. Their traditional costumes are generally made by women. To date, they still maintain the whole traditional production process from cotton planting → cotton -picking → spinning → weaving → painting → dyeing → embroidery → garment making [14, 15]. The use of dyeing plants has a broad base in the Baiku Yao community. The whole process of cotton and silk dyeing has distinct national characteristics; the process is well preserved and has very important cultural and research value. Therefore, the Baiku Yao is recognized by UNESCO as being among the nations with the most complete ethnic culture.

Like any other traditional culture, dyeing culture is also impacted by foreign culture and the market economy, which risk eliminating the traditional culture. Although it is the inevitable trend of social development that some aspects of traditional culture are replaced or automatically subside, the loss of such a well-preserved and interesting culture will be an irreparable loss to mankind. The diversity and richness of ethnic culture can provide cultural resources and dynamic support for human sustainable survival and development and should be respected and protected. In addition, as a kind of plant resource, dyeing plants play an important role in research on botanical resources. Therefore, using ethnobotanical methods, this study investigated the Baiku Yao dyeing plant resources and their traditional knowledge in Yaoshan of Guizhou and Yaozhai, Huaili village, Yaoli of Guangxi, in order to systematically record, catalog, sort, and evaluate the traditional plant dyeing folk knowledge of Baiku Yao, preliminarily analyze its mechanism, protect the inheritance and development of the Baiku Yao's traditional clothing dyeing culture, and provide materials for modern clothing color dyeing.

## Methods

### Study area

**Fig. 1:**
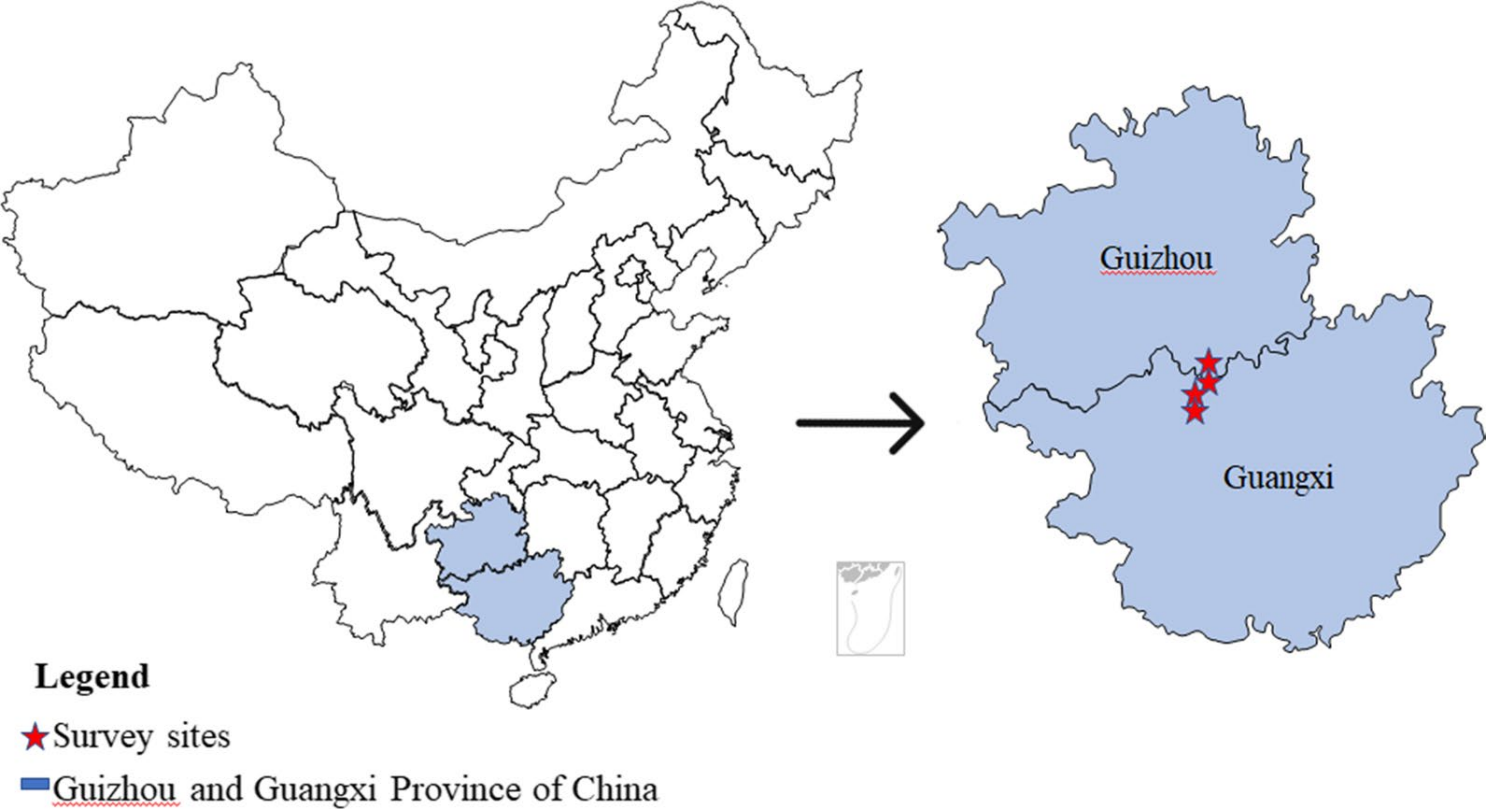
Study area location

The population of Baiku Yao numbers more than 30 thousand and is mainly distributed in Yaoshan of Guizhou and Yaozhai, Huaili, and Yaoli of Guangxi (Fig. 1); there are many mountains here amongst a small area. The area has a subtropical monsoon climate, and the territory is rich in forest vegetation [16, 17]. Most of the Baiku Yao live on the slopes of the mountainside or in the depressions at the foot of the mountain.

### Interviews and plant material collection

From January 2020 to August 2021, we investigated 61 informants (40 women and 21 men), including 2 informants aged ≥ 70, 2 informants aged 60–69, 7 informants aged 50–59, 25 informants aged 40–49, and 24 informants aged < 40. The education level of informants varied from illiterate (12 informants) to technical secondary school graduates (7 informants). Among them, 31 key informants were recommended by local village cadres, and the other 30 were selected in the local village by snowball effect. This study investigated all the plants used in the dyeing process of Baiku Yao traditional clothing, including anti-dyeing, dyeing, auxiliary dyeing, and color fixing. Free lists from each informant were elicited by the prompt: “Which plants are used for dying cotton or silk? What are the specific uses of this plant in dyeing process?” So, we can obtain a form containing order and frequency. Then, when informants listed the plant and specified uses, we would ask the main way to obtain (i.e., collect or buy), harvest season, local name, part(s) used medicinal effect, and attitude toward plant dyeing of participants (including four elements: clothing attractiveness, durability, smoothness and health care value). Next, we recorded the extraction, preparation, and dyeing process of plant dyes through participatory investigation and field investigation and collected voucher samples or took voucher photos. Through the identification of specimens and data sorting, the ethnobotanical survey results are displayed in catalogs. We collected the local traditional knowledge in accordance with the ISE Code of Ethics [18]. Permissions were provided by all participants in this study, including the local Baiku Yao people. Consent was obtained from the local communities prior to the field investigations.  The authors have all copyrights. The specimens were stored at the Guangxi Institute of Traditional Medical and Pharmaceutical Sciences.

### Statistical analyses

(1) This study used the free-list method to rank and discuss the potential value of the dyeing plants used by the Baiku Yao to guide the development and utilization of plant resources. By asking informants "What plants do you use to dye cotton and silk?", we determined list position and list frequency to assess the cognitive salience of each species [19]. Cognitive salience can represent the typicality and representativeness of each plant in Baiku Yao dyeing culture.

$$\begin{aligned} {\text{B}} & = {\text{k}} - r(i)/k - 1 \\ {\text{CS}} & = {{\left[ {\sum {B + F - 1} } \right]} \mathord{\left/ {\vphantom {{\left[ {\sum {B + F - 1} } \right]} {2Z - 1}}} \right. \kern-\nulldelimiterspace} {2Z - 1}} \\ \end{aligned}$$

k: the number of listed items, r(i): the *i*th rank of each item’s listed position; i = 1, 2, …, k. F: the number of lists in which the term is mentioned to measure the overall sample or group. Z: the number of respondents.

(2) The informant consensus factor (F_ic_) was employed to deduce whether the informant’s information was consistent when a specific plant was used to treat a particular category of ailments [20]. The theoretical value of F_ic_ ranges from 0 to 1. A higher value (close to 1) indicates that the informants agreed to use these plants for the treatment of a certain aliment. F_ic_ was calculated with the following formula:

$$F_{ic} = \frac{{N_{ur} - N_{t} }}{{N_{ur} - 1}}$$

N_ur_: the number of use reports in each category. N_t_: the number of taxa used.

## Results

### Taxonomic diversity of dyeing plants used in Baiku Yao traditional clothing

**Fig. 2:**
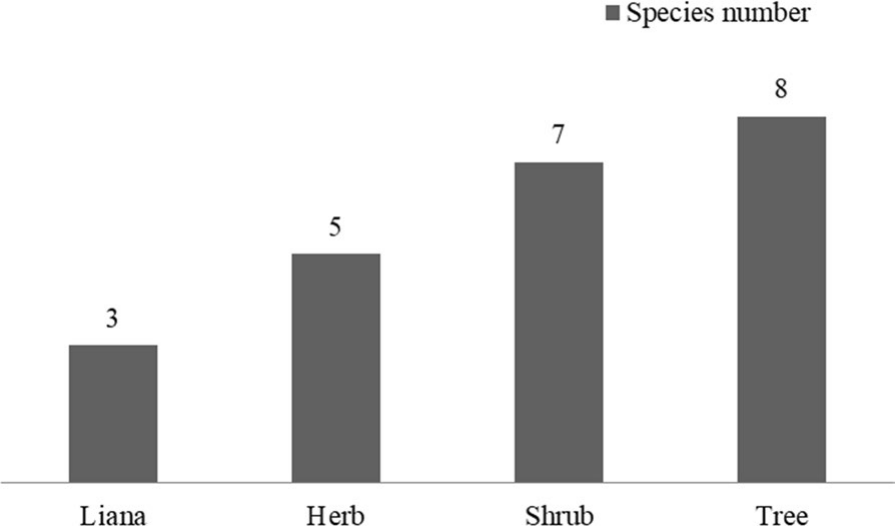
Life forms of dyeing plants used in the production of Baiku Yao traditional clothing

Through the systematic and in-depth ethnobotanical investigation of traditional clothing dyeing plants in the main area in which the Baiku Yao people live, 23 species of plants belonging to 19 families and 20 genera were recorded (see Table 1). There were 16 species (69.57%) of wild plants and 7 species (30.43%) of cultivated plants, which shows that the production and life of local people are also dependent on wild plant resources. The Baiku Yao preferred to use trees (8 species, 34.78%) and shrubs (7 species, 30.43%) and to use fewer herbs (5 species, 21.74%) and vines (3 species, 13.04%) (Fig. 2). Through the information system of Chinese rare and endangered plants (http://www.iplant.cn/rep/), 16 wild species are not endangered.

**Table 1:** Dyeing plants used in traditional costumes by the Baiku Yao nationality of China

	Family	Scientifie name	Chinese name	Yao name	Life form	Habitat	Used part(s)	Application category	Effect	Dry or fresh	Collecting time	Medicinal effect	CS	Voucher specimen	Commonly used areas
Cotton	Acanthaceae	*Strobilanthes cusia* (Nees) Kuntze	Bǎn lán 板蓝	Nong ze	Herb	Cultivated	Whole plant	Dyeing	Blue or black	Fresh	Autumn	Clearing away heat and toxic, eliminating phlegm	0.84	HRC072, HRC254	Yaoshan, Lihu, Baxu
	Dioscoreaceae	*Dioscorea cirrhosa* Lour	Shǔ liáng 薯莨	Lu ya	Liana	Wild	Tuber	Late fixing materials	Increase color and brightness	Fresh	Autumn	Having insecticidal properties	0.58	HRC98, HRC489	Yaoshan, Lihu, Baxu
	Dioscoreaceae	*Dioscorea subcalva* Prain et Burkill	Máo jiāo shǔ yù 毛胶薯蓣	Lu lu	Liana	Wild	Tuber	Late fixing materials	Increase the brightness and durability of dyed cotton cloth, and prevent fuzzing and discoloration	Fresh	Autumn	Improving blood circulation, hemostasis, relieving pain	0.31	HRC490	Yaoshan, Lihu, Baxu
	Euphorbiaceae	*Vernicia fordii* (Hemsl.) Airy Shaw	Yóu tóng 油桐	La jiao ai	Tree	Cultivated	Bark	Dyeing auxiliary	Dyeing accelerant	Dry	Autumn and winter		0.12	HRC253	Yaoshan, Lihu, Baxu
	Gleicheniaceae	*Dicranopteris pedata* (Houtt.) Nakaike	Máng qí 芒萁	Bai qing	Herb	Wild	Whole plant	Dyeing auxiliary	Dyeing accelerant	Dry	Whole year		0.05	HRC715	Lihu
	Lauraceae	*Cinnamomum parthenoxylon* (Jack) Meisner	Huáng zhāng 黄樟	Nong hai	Tree	Wild	Bark	Late fixing materials	Increase color and brightness	Dry/fresh	Autumn and winter		0.00	HRC323	Baxu
	Meliaceae	*Toona sinensis* (Juss.) Roem	Xiāng chūn 香椿	Wo you	Tree	Cultivated	Bark	Late fixing materials	Increase color and brightness	Dry/fresh	June–September	Having insecticidal properties	0.00	HRC322	Baxu
	Papilionaceae	*Mucuna birdwoodiana* Tutcher	Bái huā yóu má téng 白花油麻藤	Gu niu	Liana	Wild	Tender twigs	Late fixing materials	Increase color and brightness	Fresh	March–April	Enriching the blood, strengthening bones and tendons	0.12	HRC572	Lihu
	Poaceae	*Oryza sativa* L	Dào 稻	Cuo	Herb	Cultivated	Whole plant	Dyeing auxiliary	Enhance the adhesion of indigo	Dry	Autumn		0.25	HRC648	Yaoshan, Lihu, Baxu
	Rhamnaceae	*Rhamnus utilis* Decne	Dòng lǜ 冻绿	Nong ya	Tree	Wild	Stem	Late fixing materials	Color retention	Dry/fresh	Autumn	Aiding digestion, eliminating phlegm	0.59	HRC193, HRC334, HRC251	Yaoshan, Lihu, Baxu
	Simaroubaceae	*Ailanthus vilmoriniana* Dode	Cì chòu chūn 刺臭椿	Zhe gu zhou	Tree	Cultivated	Resin	Anti-dyeing	Prevent dye from dyeing on cotton cloth	Fresh	May–October		0.83	HRC647	Yaoshan, Lihu, Baxu
Silk	Aquifoliaceae	*Ilex chinensis* Sims	Dōng qīng 冬青	Nong ze ya	Tree	Cultivated	Leaf	Dyeing	Reddish brown	Fresh	Autumn	Having bacteriostatic properties, healing burns, treating ulcers, having hemostatic properties	0.08	HRC780	Yaoshan
	Aquifoliaceae	*Ilex kwangtungensis* Merr	Guǎng dōng dōng qīng 广东冬青	Nong ze ya	Tree	Cultivated	Leaf	Late fixing materials	Reddish brown	Fresh	May–October	Having bacteriostatic properties, healing burns, treating ulcers, having hemostatic properties	0.17	HRC502, HRC252, HRC649	Yaoshan, Lihu, Baxu
	Loganiaceae	*Buddleja officinalis* Maxim	Mì méng huā 密蒙花	Ba cuo	Shrub	Wild	Flower	Dyeing auxiliary	Yellow	Dry/fresh	Autumn	Lowering blood pressure,having hemostatic properties,reducing swelling	0.06	HRC645	Yaoshan, Lihu, Baxu
	Melastomataceae	*Melastoma dodecandrum* Lour	Dì niè 地菍	~	Herb	Wild	Whole plant	Dyeing auxiliary	Toning	Fresh	Autumn		0.00	HRC781	Yaoshan
	Meliaceae	*Chukrasia tabularis* A. Juss	Má liàn 麻楝	~	Tree	Wild	Bark	Dyeing auxiliary	Reddish brown	Dry/fresh	May–June	Stimulating menstrual flow, relieving pain	0.02	HRC74	Lihu
	Myrsinaceae	*Myrsine semiserrata* Wall	Zhēn chǐ tiě zǐ 针齿铁仔	Nong cuo	Shrub	Wild	Stem and leaf	Dyeing	Reddish brown	Fresh	May–October	Clearing away heat and toxic	0.00	HRC487	Baxu
	Rhamnaceae	*Rhamnus utilis* Decne. var. *utilis*	Dòng lǜ 冻绿	Nong ya	Tree	Wild	Stem	Dyeing	Reddish brown	Dry/fresh	Autumn	Aiding digestion, eliminating phlegm	0.59	HRC193, HRC334, HRC251	Yaoshan, Lihu, Baxu
	Rubiaceae	*Gardenia jasminoides* Ellis	Zhī zǐ 栀子	Gua nong	Shrub	Wild	Fruit	Dyeing	Yellow	Dry/fresh	Autumn and winter		0.09	HRC482, HRC250	Yaoshan, Lihu, Baxu
	Rutaceae	*Murraya euchrestifolia* Hayata	Dòu yè jiǔ lǐ xiāng 豆叶九里香	Dong zai	Shrub	Wild	Leaf	Late fixing materials	Reddish brown	Fresh	Autumn	Eliminating wind and removing dampness, relieving pain	0.07	HRC646	Lihu
	Symplocaceae	*Symplocos lucida* (Thunb.) Sieb. et Zucc	Guāng liàng shān fán 光亮山矾	Nong ze ya	Shrub	Wild	Leaf	Late fixing materials	Reddish brown	Fresh	May–October		0.15	HRC672	Lihu
	Theaceae	*Eurya groffii* Merr	Gǎng líng 岗柃	Nong cuo wa	Shrub	Wild	Leaf	Late fixing materials	Reddish brown	Fresh	May–October	Eliminating phlegm, relieving cough, reducing swelling	0.06	HRC488	Baxu
	Theaceae	*Eurya tetragonoclada* Merr. et Chun	Sì jiǎo líng 四角柃	Nong cuo wa	Shrub	Wild	Leaf	Dyeing auxiliary	Reddish brown	Fresh	May–October	Clearing away heat and toxic, nourishing the liver to improve visual acuity	0.00	HRC483	Baxu
	Zingiberaceae	*Curcuma longa* L	Jiāng huáng 姜黄	Gang guai	Herb	Wild	Rhizome	Dyeing	Yellow	Fresh	Autumn and winter	Stimulating menstrual flow, relieving pain	0.04	HRC552	Yaoshan, Lihu, Baxu

**Fig. 3:**
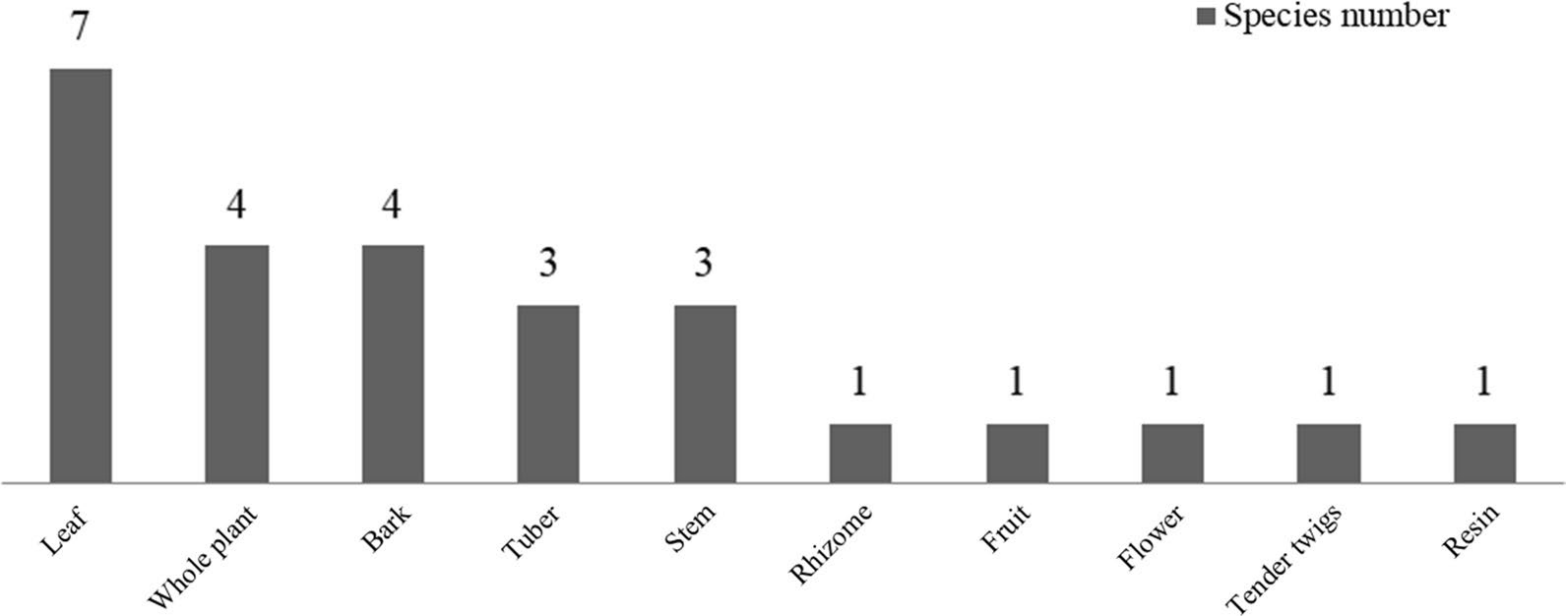
Parts of dyeing plants used in the production of Baiku Yao traditional clothing

Of the plant species used to dry cotton or silk, several plant part(s) are used, including leaves (7 species), whole plants (4 species), tubers, bark and stems (3 species, respectively), fruit, peel, flowers, twigs and resin (1 species, respectively), as shown in Fig. 3. Baiku Yao peoples’ selection of traditional clothing dyeing plants did not focus on certain families and genera or on specific plant parts, which reflected the very rich source of dyeing plants used, their in-depth understanding of the surrounding plants and plant properties, and accumulated rich traditional knowledge in the long-term dyeing process.

### Traditional knowledge of plant dyeing

#### Traditional knowledge of cotton dyeing

The ethnobotanical survey showed that the Baiku Yao dyed cotton with 11 plant species. According to their applications, the 11 plants are divided into four types: anti-dyeing, dyeing, dyeing auxiliary and late fixing materials; only 1 species is used for anti-dyeing and dyeing materials, 3 species are used for dyeing auxiliary materials, and 6 species are used for later fixing materials.

The Baiku Yao's clothing is mainly composed of cotton. The whole process of cotton planting, cotton picking, spinning, weaving, painting, dyeing, color fixation, paste removal, secondary dyeing and color fixation was completed independently by Baiku Yao women. The color of the garments is mainly composed of blue and black. Through unique anti-dyeing technology, there are patterns of various shapes on the clothing. The investigation results show that the dyeing process of Baiku Yao cotton cloth mainly includes the following 6 steps:

**Step 1: Pasting and drawing**

**Fig. 4:**
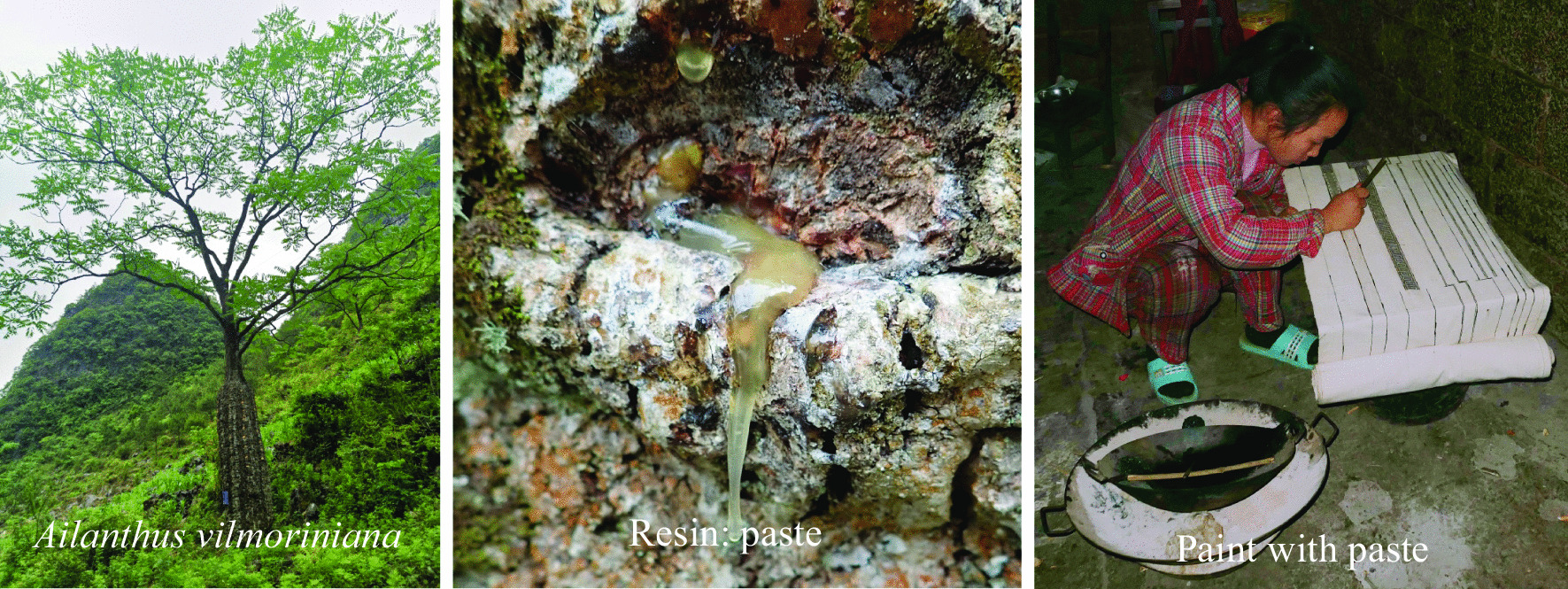
Step 1: Pasting and drawing with the resin of *Ailanthus vilmoriniana*

The key is that Baiku Yao women skillfully use the resin of *Ailanthus vilmoriniana* as an anti-dyeing material (Fig. 4). The sticky paste is made of *Ailanthus vilmoriniana* resin taken during May every year. The paste is melted in a pot at approximately 70 ℃ from October to November, and an appropriate amount of butter is added and stirred after melting. The mixture is filtered into a basin while it is hot. A painting knife is dipped into the melted paste, and various patterns are drawn on the white cotton cloth (Fig. 4). The remaining paste is solidified after cooling and stored for later use.

**Step 2: Dyeing with indigo**

**Fig. 5:**
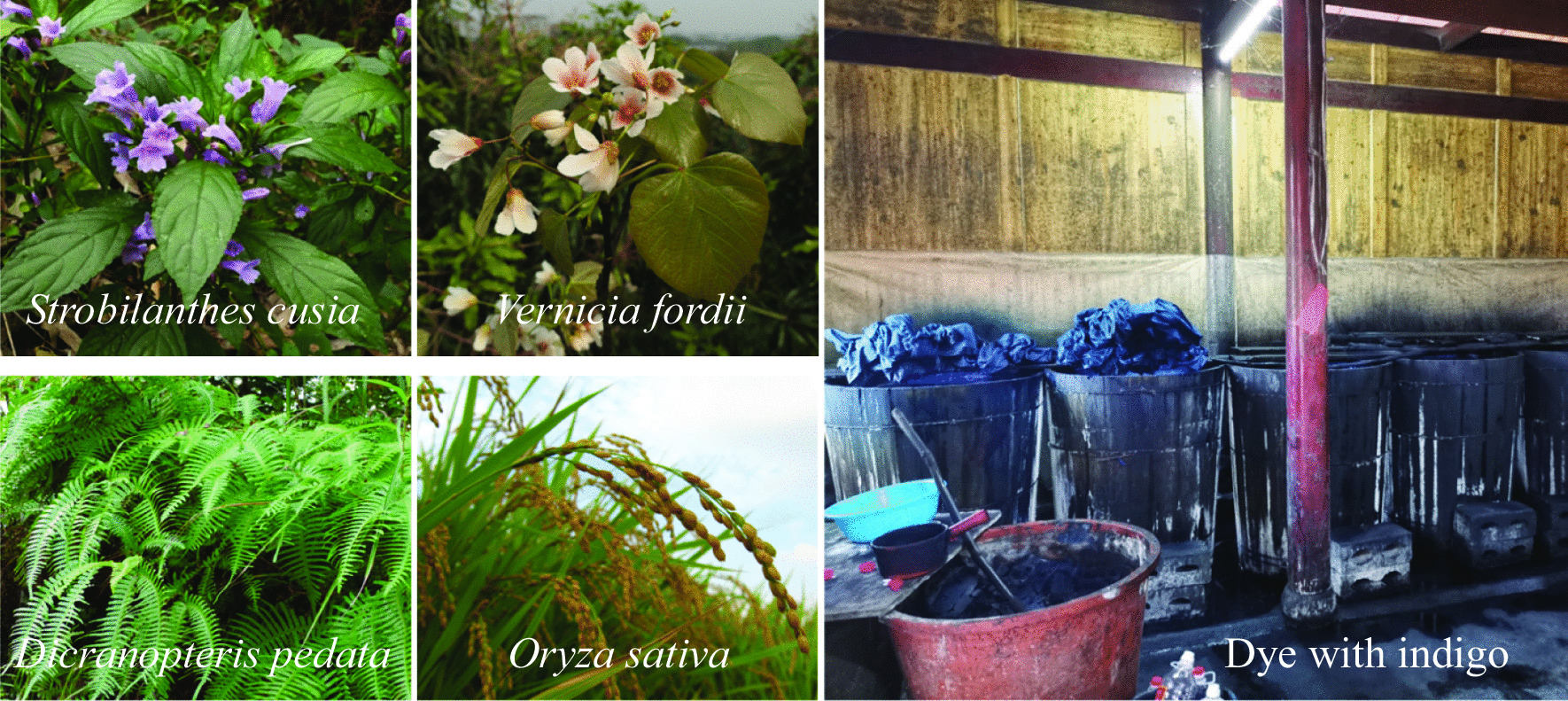
The plants used for making dyeing liquor and the scene dyed with indigo

The indigo paste made by *Strobilanthes cusia* is mixed with the peel of *Vernicia fordii*, *Dicranopteris pedata* or the ash from the burning of *Oryza sativa* to make dyeing liquor (Fig. 5). It is then held for approximately 10 days according to the proportions of “90 kg water, 0.5 kg wine and 0.5 kg indigo paste”, and whether the dyeing water turns yellow-green is observed. On a clear day, the cotton cloth painted with sticky paste is placed into the dye vat, soaked for 2 h, taken out while the mixture is replenished, and then put it back into the dye vat. The cloths are dyed 3–4 times a day, washed and dried for 5–6 consecutive days. Items are considered dyed well after 5 cycles.

**Step 3: Fixing the color and brightening the cotton cloth**

**Fig. 6:**
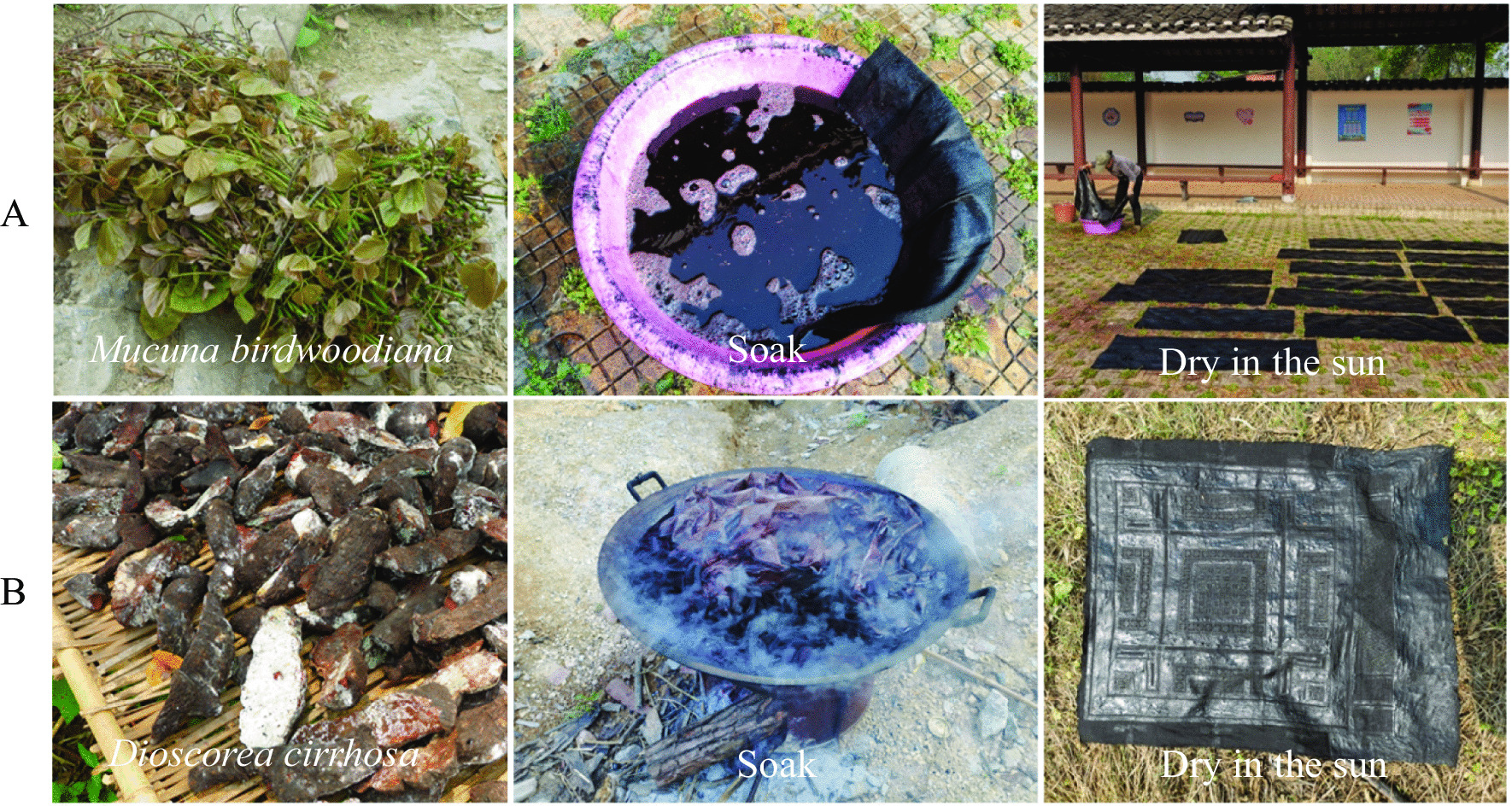
Two main methods of fixing the color and brightening the cotton cloth (**A** represent “Ji Xue Teng dye”; **B** represent “Shu Liang dye”)

There are two main methods: (1) From March to May every year, local people will go up the mountain to collect the tender twigs of *Mucuna birdwoodiana*, mash the tissues and collect the juice, put the cloth dyed with indigo into the juice of *Mucuna birdwoodiana*, take it out and dry it after soaking, and repeat this 7–8 times a day for 2–3 days. This method is common in Lihu and Baxu. The local people commonly call it “Ji Xue Teng dye” (Fig. 6A). (2) The second method is common in Yaoshan, Lihu and Baxu. It is commonly known as "Shu Liang dye" by locals (Fig. 6B). In July of the lunar calendar every year, the locals will go up the mountain to collect *Dioscorea cirrhosa*, boil slices of the material until the water is dark red, soak the cotton cloth in the water and dry it, and repeat 7–8 times a day for 2–3 consecutive days. However, in order to make the cotton cloth red and look more beautiful, most Baiku Yao in Baxu township will add old stems of *Rhamnus utilis* in the dyeing process, and a few people will also add the old stem skin of *Toona sinensis* or *Cinnamomum camphora*.

**Step 4: Removing the paste**

**Fig. 7:**
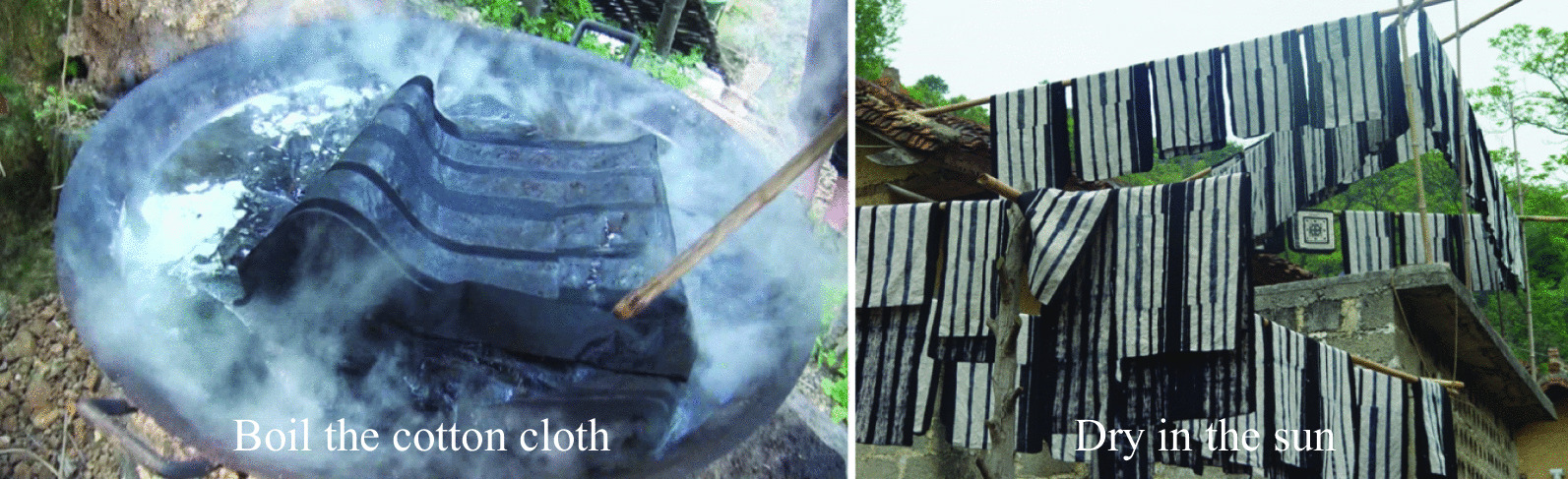
The process of removing the paste

In April of the lunar calendar, the local people boil the cotton cloth in water made of ash from the burning of *Oryza sativa*. During the cooking process, the cotton cloth is continuously turned so that the paste can drip off evenly. Then, the cloth is removed, washed with water and dried in the sun. The black-and-white pattern of the cotton cloth will be clearly visible (Fig. 7).

**Step 5: Dyeing with indigo again**

The cotton cloth is placed into indigo water for approximately 3 min (the purpose is to dye it light blue or dark blue according to personal preference), and then removed. The white area is then dyed blue, and the cloth is washed with clean water and dried in the sun.

**Step 6: Fixing the color again and brightening the cotton cloth**

**Fig. 8:**
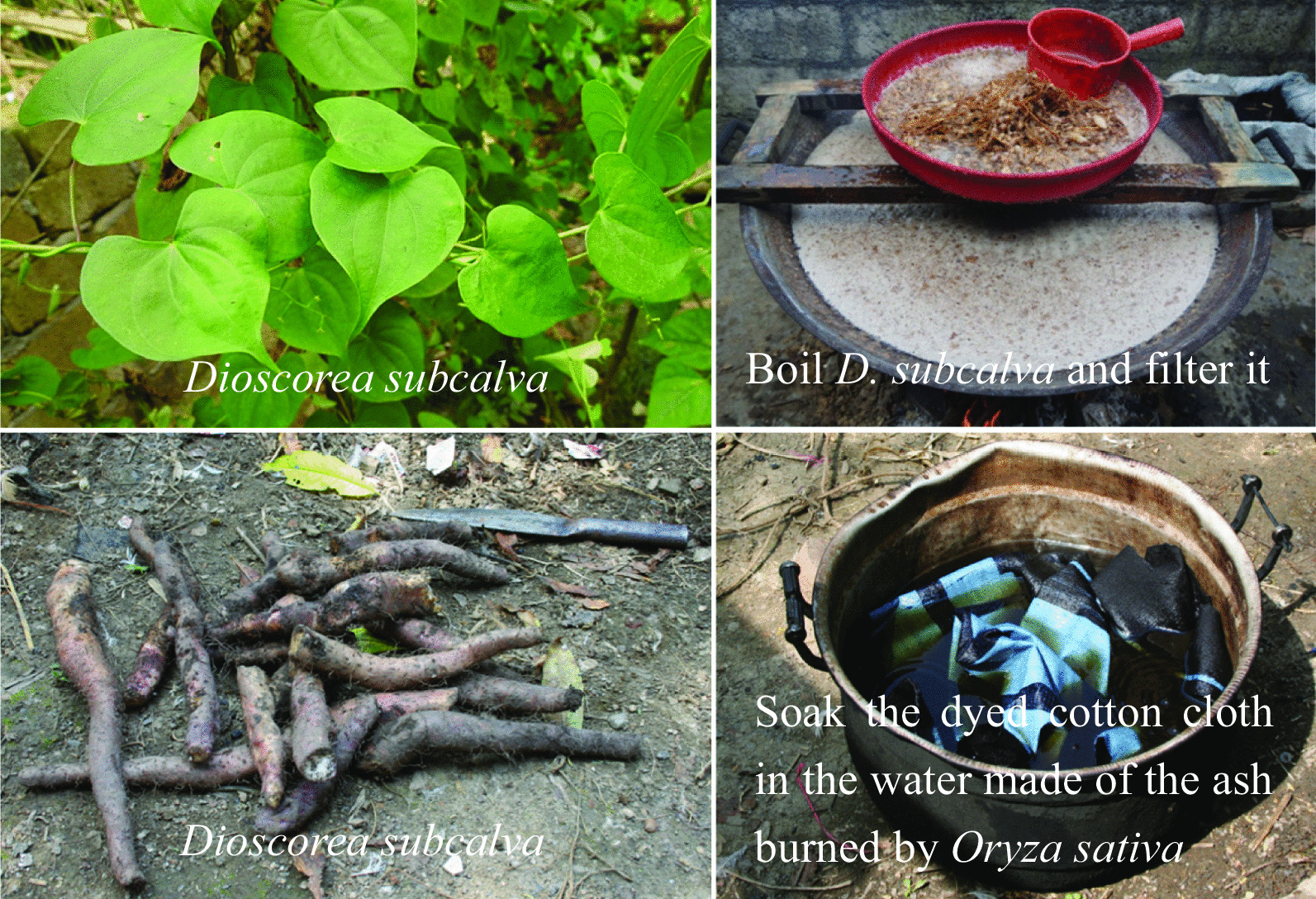
Fixing the color again and brightening the cotton cloth

The water made of the ash from the burning of *Oryza sativa* is filtered, the dyed cotton cloth is placed in it and then dried in the sun. Then, the juice from boiling *Dioscorea subcalva* is poured onto the cloth until it is completely soaked (Fig. 8), and the cloth is again dried in the sun. The purpose of this is to make the cotton cloth brighter and more durable, and it does not easily become fuzzy or decolorized.

#### Traditional knowledge of silk dyeing

**Fig. 9:**
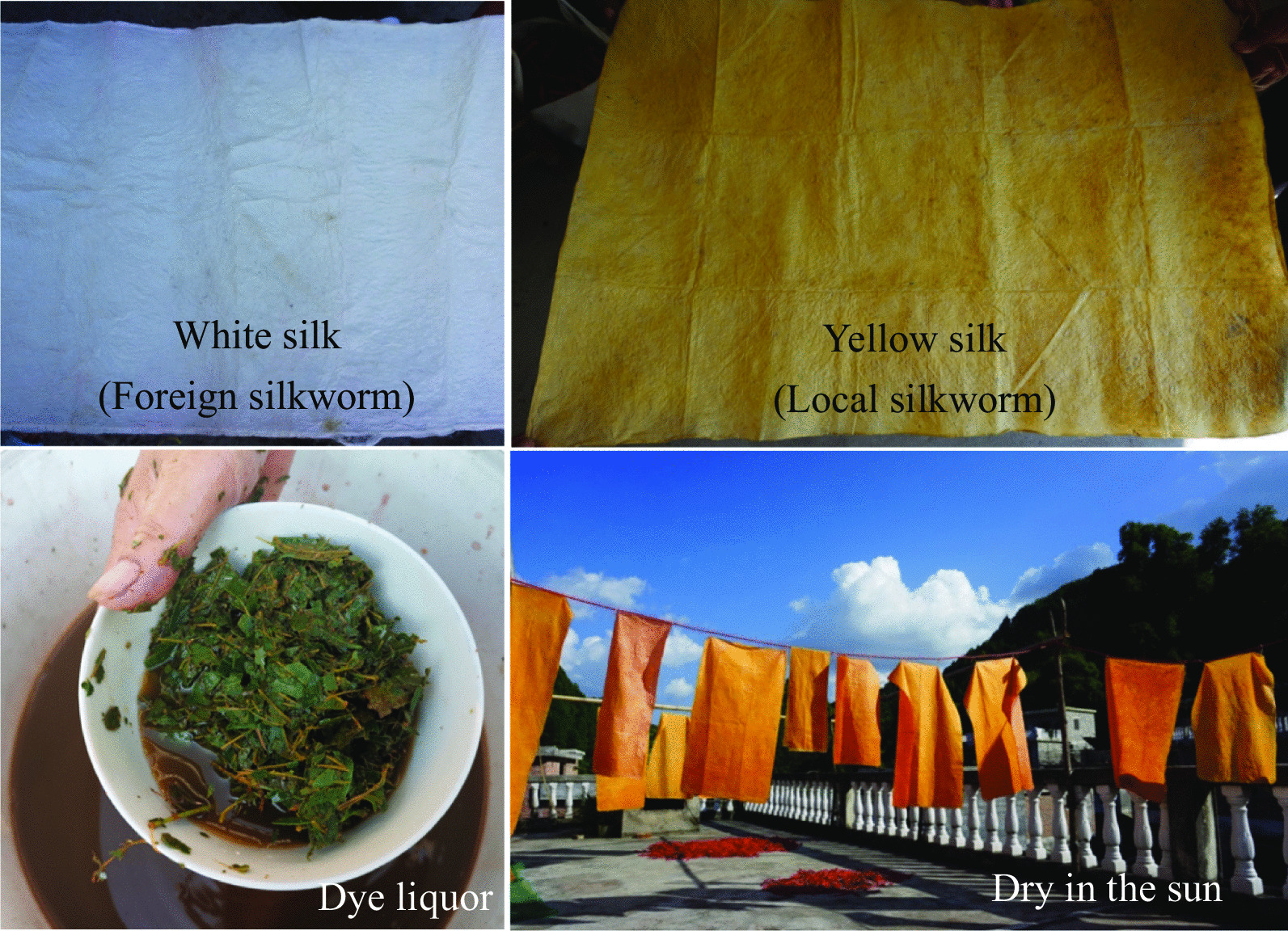
Both kinds of silk cloth were dyed reddish brown by Baiku Yao people

The Baiku Yao usually dye two kinds of silk: that from local silkworms which produce yellow silk and foreign silkworms which produce white silk. Locally, whether white silk or yellow silk is present, the silk cloth is finally dyed reddish-brown (Fig. 9). The Baiku Yao dye silk with 13 plant species.

**Local silkworm**: Baiku Yao people living in different places have different methods of making dye liquor (Fig. 9). All informants mentioned *Rhamnus utilis*. In Yaoshan, local people boil the stems of *Rhamnus utilis* until an orange-yellow color is apparent. After cooling, the mashed juice of *Ilex Chinensis* is added, the mashed juice of *Melastoma dodecandrum* is added, and the sample finally becomes reddish-brown. In Lihu, local people mix the water from the boiled stems of *Rhamnus utilis* with the mashed juice of the leaves of *Symplocos Lucida* or *Ilex kwangtungensis* and then add the mashed juice from the leaves of *Murraya euchrestifolia* or the stem skin of *Chukrasia tabularis* to make the dye liquor reddish brown. In Baxu, local people first mix the water from the boiled stems of *Rhamnus utilis* with the mashed juice from the leaves of *Ilex kwangtungensis* and then mix the mashed juice from the leaves of *Eurya groffii* or *Eurya tetragonoclada* and *Myrsine semiserrata* to make the dye liquor reddish brown. In July of the lunar calendar every year, people soak the yellow silk in the above dyeing liquor for 3–4 min, take it out and dry it in the sun, and repeat the process of “soaking-drying” 5–6 times until the silk is dyed reddish-brown. The number of repetitions will vary according to personal preference for color.

**Foreign silkworm**: People dye white silk into yellow first, and then dye yellow into reddish-brown. Plant parts that can produce yellow dye include the rhizomes of *Curcuma longa*, the fruits of *Gardenia jasminoides*, and the flowers of *Buddleja Officinalis*. According to different seasons, people choose one of the above plants to make yellow dye liquor. The dyeing process is relatively simple. The plants are crushed and soaked in water. The water is heated until the water changes color, and the cloth is soaked for 3 min, removed, and dried in the sun; this is repeated for 4–5 times until the cloth is dyed reddish-brown according to the local silkworm dyeing method.

### Evaluation and screening of dyeing plants used in the production of Baiku Yao traditional clothing

**Fig. 10:**
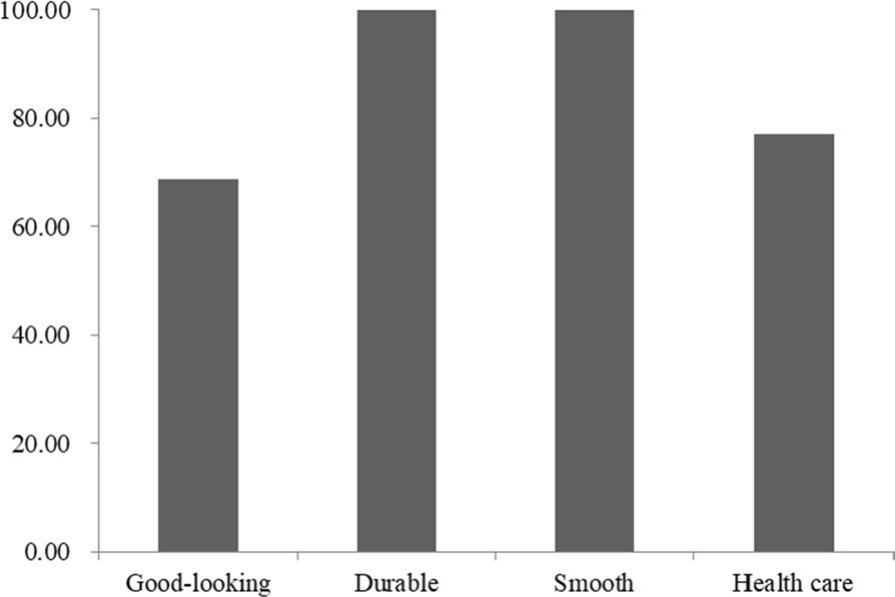
The attitude of the Baiku Yao informants toward plant dyeing

To understand the Baiku Yao's motivation to use plants for dyeing, 41 informants were interviewed (Fig. 10). The results showed that all informants agreed that the dyed fabric was durable and smooth and did not hurt the skin. A total of 68.85% of informants thought that the color of traditional Baiku Yao costumes was attractive, and 77.05% of informants thought that wearing plant-dyed clothes could treat and prevent some skin diseases.

In this study, the CS values of these 23 dyed plants were ranked based on the free-list method. The higher the score, the greater the potential research value of the plant. The plants with CS > 0.8 were *Strobilanthes cusia* and *Ailanthus vilmoriniana*. The plants with 0.5 < CS < 0.8 were *Rhamnus utilis* and *Dioscorea cirrhosa*. The plants with 0.1 < CS < 0.5 included *Dioscorea subcalva*, *Oryza sativa*, *Ilex kwangtungensis*, *Symplocos Lucida*, *Mucuna birdwoodiana*, and *Vernicia fordii*. The CS values of other plants were less than 0.1 (Table 2).

**Table 2:** The CS values of dyed plants

Latin name	B value	Nm	Frequency (F)	Cognitive salience (CS)
*Strobilanthes cusia*	47.21	55	0.90	0.84
*Ailanthus vilmoriniana*	43.88	57	0.93	0.83
*Rhamnus utilis*	25.16	47	0.77	0.59
*Dioscorea cirrhosa*	21.81	49	0.80	0.58
*Dioscorea subcalva*	6.70	32	0.52	0.31
*Oryza sativa*	4.93	26	0.43	0.25
*Ilex kwangtungensis*	7.95	14	0.23	0.17
*Symplocos lucida*	6.72	13	0.21	0.15
*Mucuna birdwoodiana*	3.03	13	0.21	0.12
*Vernicia fordii*	1.91	14	0.23	0.12
*Gardenia jasminoides*	2.93	9	0.15	0.09
*Ilex chinensis*	3.29	7	0.11	0.08
*Murraya euchrestifolia*	3.05	6	0.10	0.07
*Buddleja officinalis*	1.32	7	0.11	0.06
*Eurya groffii*	2.98	5	0.08	0.06
*Dicranopteris pedata*	0.55	7	0.11	0.05
*Curcuma longa*	0.36	6	0.10	0.04
*Chukrasia tabularis*	1.00	2	0.03	0.02
*Eurya tetragonoclada*	0.45	1	0.02	0.00
*Melastoma dodecandrum*	0.43	1	0.02	0.00
*Toona sinensis*	0.18	1	0.02	0.00
*Cinnamomum parthenoxylon*	0.09	1	0.02	0.00
*Myrsine semiserrata*	0.00	1	0.02	0.00

### There is homology between dyeing plants and medicinal plants

The ethnobotanical survey results showed that the Baiku Yao informants believe that 15 dyeing plants also have medicinal value (Table 3). Informants reported that 15 species can prevent or alleviate illnesses in 18 ailment categories. In nine ailment categories (enriching the blood, strengthening bones and tendons, aiding digestion, improving blood circulation, eliminating wind and removing dampness, stimulating menstrual flow, lowering blood pressure, nourishing the liver to improve visual acuity, and relieving cough), informants reported using only one plant species; so the Fic values for these uses is 1.00. Aside from these nine categories, a higher F_ic_ value (> 0.7) was cited for clearing away heat and toxic materials, eliminating phlegm, relieving pain, reducing swelling, and having bacteriostatic properties.

**Table 3:** The F_ic_ values of plants treating 18 ailment categories

Ailment categories	Plant species	N_t_	N_ur_	F_ic_
Enriching the blood	*Mucuna birdwoodiana*	1	5	1.00
Strengthening bones and tendons	*Mucuna birdwoodiana*	1	6	1.00
Aiding digestion	*Rhamnus utilis*	1	13	1.00
Improving blood circulation	*Dioscorea cirrhosa*	1	5	1.00
Eliminating wind and removing dampness	*Murraya euchrestifolia*	1	3	1.00
Stimulating menstrual flow	*Curcuma longa*	1	2	1.00
Lowering blood pressure	*Gardenia jasminoides*	1	6	1.00
Nourishing the liver to improve visual acuity	*Buddleja officinalis*	1	2	1.00
Relieving cough	*Eurya groffii*	1	3	1.00
Clearing away heat and toxic material	*Strobilanthes cusia, Melastoma dodecandrum, Buddleja officinalis, Chukrasia tabularis*	4	61	0.95
Eliminating phlegm	*Rhamnus utilis, Eurya groffii*	2	16	0.93
Relieving pain	*Dioscorea cirrhosa, Curcuma longa, Murraya euchrestifolia*	3	12	0.82
Reducing swelling	*Strobilanthes cusia, Gardenia jasminoides, Eurya groffii*	3	12	0.82
Having bacteriostatic properties	*Ilex chinensis, Ilex kwangtungensis*	2	5	0.75
Healing burns	*Ilex chinensis, Ilex kwangtungensis*	2	4	0.67
Treating ulcers	*Ilex chinensis, Ilex kwangtungensis*	2	4	0.67
Having hemostatic properties	*Dioscorea cirrhosa, Ilex chinensis, Melastoma dodecandrum, Gardenia jasminoides, Ilex kwangtungensis*	5	12	0.64
Having insecticidal properties	*Toona sinensis, Cinnamomum parthenoxylon*	2	2	0.00

## Discussion

### The diversity and uniqueness of traditional knowledge of dyeing Baiku Yao national clothing

All minority nationalities have unique cultural traditions that are different from those of other nationalities. As the most direct and convenient visual symbol to convey information, color has a profound impact on human production and life; in other words, the color cultures of different nationalities exhibit diversity and uniqueness, which is reflected in the dyeing purposes, hues, dyeing plant species, plant part(s) used and traditional dyeing processes [21].

The Baiku Yao cotton and silk dyeing processes have national characteristics and novelty as well as important research value. The Baiku Yao use only one kind of dyeing material (*Strobilanthes cusia*) in the cotton dyeing process, use unique anti-dyeing material (resin of *Ailanthus vilmoriniana*), three kinds of auxiliary dyeing materials (*Oryza sativa*, *Dicranopteris pedata*, and *Vernicia fordii*), and make full use of the rich plant species (*Dioscorea cirrhosa*, *Rhamnus utilis*, *Mucuna birdwoodiana*, *Toona Sinensis*, *Cinnamomum parthenoxylon*, and *Dioscorea subcalva*) for secondary color fixation and brightening. Such unique technology makes Baiku Yao clothing diverse, beautiful, durable, and not easily faded or fuzzy. The Baiku Yao uses 6 kinds of dyeing materials (*Rhamnus utilis*, *Ilex chinensis*, *Curcuma longa*, *Melastoma dodecandrum*, *Myrsine semiserrata*, and *Gardenia* *jasminoides*) in the process of silk dyeing. Because Baiku Yao women prefer reddish-brown colors, they will combine a variety of plants to ultimately dye the silk reddish brown. In addition, the Baiku Yao uses numerous dyeing plants to dye silk. The reason is that silk fiber is a protein fiber with a strong affinity for plant dyestuff and good dyeing performance, allowing a more convenient and trouble-saving dyeing process. However, the heat resistance of silk is slightly poor, so Baiku Yao people will not dye silk at high temperature in the traditional dyeing process. However, cotton fiber has good heat resistance and can withstand high-temperature treatment for a short time.

The utilization of dyeing plants has a strong relationship with ethnic culture. The study of traditional dyeing plants is helpful to understand the relationship between specific cultures and plants [3, 5, 9, 22]. *Ailanthus vilmoriniana* generally grows around Baiku Yao village. To date, *A. vilmoriniana* has not been found in other ethnic villages. The wisdom to use the resin of *A. vilmoriniana* is unique to the Bai Ku Yao; if these pastes from *A. vilmoriniana* were unavailable, the Baiku Yao would never be able to make colorful national clothing. To date, the role of paste has not been replaced by any chemicals. Moreover, the Baiku Yao know how to protect *A. vilmoriniana*. People must cut and chisel the trunk with sharp knives and axes every year. If there is a break in the middle of a year, the tree will die. Baiku Yao people cut and care for *A. vilmoriniana* trees year after year and never use them for other purposes. Ethnic culture is conducive to the sustainable utilization of plant resources. Because the trunk of *A. vilmoriniana* is large in the middle and small at both ends, similar to a pregnant woman, many people also call it the mother tree of Baiku Yao. Baiku Yao's technique of dyeing silk into reddish-brown using *Chukrasia tabularis*, *Eurya tetragonoclada*, *Myrsine semiserrata*, *Murraya euchrestifolia*, *Eurya groffii*, *Symplocos Lucida*, and *Ilex kwangtungensis* is very unique. This use of these species is reported here for the first time, reflecting the diversity and uniqueness of the Baiku Yao's mastery of dyeing plants for production of their traditional clothing. However, other ethnic groups in China have less preference for reddish brown, so there are fewer plants known to produce this color.

Members of the same nation may live in different regions and face different plant resources. Thus, the dyeing plants and related traditional knowledge are also different. Local villagers choose plants that are easier to collect according to local conditions. For example, *Mucuna birdwoodiana* is planted only in Lihu and not in other places. The Baiku Yao people in Yaoshan, Lihu, and Baxu use *Dioscorea subcalva* to fix and brighten cotton in the later stage, while the Baiku Yao people in Baxu use *Toona Sinensis* or *Cinnamomum parthenoxylon* instead. “Ji Xue Teng dye” is only well known by the people living in Baxu and Lihu.

### The potential value of dyeing plants used in Baiku Yao traditional clothing

With the continuous increases in mass consumption, people have higher expectations for their quality of life. Plant-based dyeing has won favor because of its natural color, insect prevention, sterilization and reduction of the harms from chemical processing to the human body [4, 23].

This study evaluated the potential value of each dyeing plant based on the CS value. The results show 10 plants with high scores, such as *Strobilanthes cusia*, *Ailanthus vilmoriniana*, *Rhamnus utilis*, *Dioscorea cirrhosa*, *Dioscorea subcalva*, *Oryza sativa*, *Ilex kwangtungensis*, *Symplocos lucida*, *Mucuna birdwoodiana* and *Vernicia fordii*. ***Strobilanthes cusia***: as a kind of "bluegrass", *S. cusia* is an important industrial crop in Chinese history. It is not only the main dyeing material used by Baiku Yao in dyeing cotton cloth but also used by many nationalities and regions to prepare indigo paste for dyeing [3, 12, 24]. Moreover, studies have reported that indigo paste has antibacterial, antiviral and other health effects [25, 26]. ***Ailanthus vilmoriniana***: Baiku Yao women skillfully use the resin of *A. vilmoriniana* as an anti-dyeing material to draw exquisite pictures. However, it was first reported to be used in the dyeing process and has high development value. ***Rhamnus utilis***: *R. utilis* is a very important green dye in Chinese history [27, 28]. It is also an indispensable plant in Baiku Yao's traditional cotton and silk dyeing, and its use frequency was very high. However, Baiku Yao people use the peeled old stems of *R. utilis* as later color fixing materials to preserve the black color of cotton. ***Dioscorea cirrhosa***: China's "fragrant cloud gauze" was selected in the second round of national intangible cultural heritage sites in 2008. *D. cirrhosa* is used as a natural plant dye [29]. Its chemical composition is mainly tannins, which have certain antibacterial [29] and anti-ultraviolet effects [30]. However, the tubers of *D. cirrhosa* were used by Baiku Yao people as color fixing materials in the later stage to increase the chromaticity and brightness, and they also use the tubers of *D. cirrhosa* to kill insects. ***Dioscorea subcalva***: Baiku Yao people often use tubers of *D. subcalva* to increase the brightness and durability of cotton cloth and prevent cotton cloth from fuzzing and discoloring. Moreover, they believe that *D. subcalva* has the effects of improving blood circulation and hemostasis and relieving pain, marking the first reports indicating that *D. subcalva* has the potential for further research and development. ***Oryza sativa***: When making dyeing liquor for cotton cloth, people often add the ash resulting from the burning of *Oryza sativa* to increase the adhesion of indigo paste and the color of dyed cotton cloth. The reason may be that *Strobilanthes cusia* contains indican, which is not considered a glycoside. It is an ester generated from indolylmethanols and fructuronic acid. The ester bond can be broken when it meets plant ash (an alkaline solution), and indole alcohol can be hydrolyzed to form indigo [31]. ***Ilex***: There are two species of *Ilex* used by the Baiku Yao people for dyeing silk: *Ilex kwangtungensis*, which is mainly used for later color fixation, and *Ilex chinensis*, which is used to dye silk reddish brown. There are also relevant literature reports that silk can be dyed reddish brown with fallen leaves of *I. chinensis* [32, 33], but Baiku Yao people prefer to dye silk with its fresh leaves. Local people believe that *I. kwangtungensis* and *I. chinensis* have certain antibacterial and hemostatic effects and can heal burns and treat ulcers. It has been reported that the genus *Ilex* contains triterpenoids, flavonoids and other chemical components, which have anti-inflammatory and antibacterial activities [34]; it is necessary to perform some research on other active and fixed color principles in the future. ***Symplocos lucida***: There are no relevant studies on *S. lucida* used in the late fixation of silk dyeing. ***Mucuna birdwoodiana***: This species is mainly used for fixing the color of cotton cloth in the later stage. People pointed out that it is not only used for dyeing but also as a traditional Chinese medicinal crop that can strengthen bones and tendons (N_ur_ = 6) and enrich the blood (N_ur_ = 5), which has been supported by the literature. *M. birdwoodiana* can significantly promote hematopoiesis in mice [35, 36]. ***Vernicia fordii***: This species is a crop with high economic value. China is the main exporter of wood oil extracted from *V. fordii*, and its peel can be used to extract potassium carbonate (K_2_CO_3_) to make activated carbon. Baiku Yao people often use the peel of *V. fordii* as the dyeing catalyst when dyeing cotton cloth to speed up the dyeing process; the reason may be that indigo is a reducing dye and can only be dissolved under alkaline conditions, while the peel of *V. fordii* contains K_2_CO_3_. The CS value of other plants is relatively low, and there is also a lack of relevant research or evidence to show their dyeing functions. Further experiments are needed to verify this traditional knowledge and fully develop and utilize dyeing plants. When screening important dyeing plants, compared with modern science and technology, ethnobotany has unexpected benefits, and can save major human, financial and material resources in the evaluation of the safety, effectiveness and durability of dyeing plant resources.

Some species are also widely used as food colorants, such as *Curcuma longa*, *Gardenia jasminoides* and *Buddleja officinalis* [37–39]. Thus, why are their CS values very low in this study? Why do only a few of the 61 informants recognize that they have development potential? First, we believe that *Curcuma longa*, *Gardenia* *jasminoides*, and *Buddleja officinalis* are often used as food pigments, and their frequency in silk dyeing is not very high; for example, Bai people use *C. longa*, *G.* *jasminoides*, and *B. officinalis* to dye cloth [16], while Baiku Yao uses them to dye silk. Second, there are differences between different ethnic cultures, and the utilization of plant resources is different. For example, nation A recognizes a plant as an important and precious resource, but nation B ignores it.

In particular, 15 plant species were considered by local people to have certain medicinal value, which not only shows the importance of these plants in fiber coloring but also shows that they have drug and health care functions. Therefore, from the perspective of environmental protection and human health, textiles dyed with plant dyes may become commonly used in close-fitting clothes, such as infant products, health underwear, silk scarves, summer clothes, pajamas, and bedding [40]. In this regard, it is necessary to further study the mechanism of plant pigments in the treatment of diseases and apply these materials scientifically to human health care.

### We should strengthen the protection of traditional knowledge of dyeing plants used in Baiku Yao traditional clothing

The Baiku Yao people have accumulated rich and unique traditional knowledge of plant-based cloth dyeing. This traditional knowledge is mainly in the hands of women. The inheritance is mainly from mother to daughter. There are also frequent exchanges between neighbors. They often work together to dye cotton or silk. The Baiku Yao men hardly participate in the production of traditional clothing. Although some Baiku Yao men know the use of a small amount of dyeing plants, they do not know the use method. The dyeing processes of the Baiku Yao can be inherited in a relatively complete form together with their ethnic culture. On the one hand, this is because the Baiku Yao agree upon their own ethnic dyeing culture. On the other hand, the Baiku Yao belong to a nation with the same roots and origins; they mainly live in the mountainous areas of Nandan County of Guangxi and Libo County of Guizhou, and foreign culture has little impact here [30]. However, with the development of society, young people go out to work, and plant dyeing is time-consuming and laborious. Young Baiku Yao women are more interested in embroidery, the ability to master plant dyeing is becoming increasingly weaker, resulting in a great threat to the inheritance of the Baiku Yao traditional clothing dyeing process. At present, much traditional knowledge is facing the risk of disappearing. However, we believe that the disappearance of traditional knowledge related to plant dyeing is not an inevitable trend because the Baiku Yao's understanding, discovery, and utilization of dyeing plants have the dual characteristics of being based on their ethnic culture and the surrounding biogeographical flora: (1) indigenous culture is an important part of world civilization. Ignoring, belittling, or even disdaining the indigenous culture and unrealistically forcing the implementation of so-called modern civilization will destroy the natural ecosystem and traditional knowledge on which Baiku Yao peoples depend, aggravate the dependence of Baiku Yao communities on external society, and lead to the reduction and loss of biodiversity of Baiku Yao communities. (2) Dyeing plants play an important role in the survival and development of local people. They are also an important component of the local flora and maintain its integrity [41]. To realize the effective protection and sustainable utilization of biological resources, we think that the printing and dyeing industry, plant resource developers, and ethnic culture researchers should work together to find new natural, safe and healthy dyeing plants and determine key plant resources on the basis of systematic investigation, complete records, scientific evaluation, and in-depth research. The application of traditional knowledge will make a significant contribution to biodiversity conservation and sustainable socio-economic development.

## Conclusion

Dyeing plants provide the material basis for the colors favored in Baiku Yao clothing culture and have high utilization and protection value. Ethnic culture also promotes and strengthens the utilization and protection of dying plants. Some dyeing plants used by the Baiku Yao are also medicinal plants. These plants not only meet the color needs of the Baiku Yao people but also have health care effects, which is in line with recent trends in demand for green dyeing materials and health products among modern people. This study will aid in finding new natural, safe and healthy textile dyes, and it is of positive significance in promoting regional economic development, national cultural inheritance, and biodiversity protection.

## Data Availability

We have already included all data in this manuscript.
